# Utility of Cone-Beam Computed Tomography in the Detection of Low Bone Mass—A Systematic Review

**DOI:** 10.3390/jcm12185890

**Published:** 2023-09-11

**Authors:** Ioana Ruxandra Poiana, Ramona Dobre, Razvan-Ionut Popescu, Silviu-Mirel Pituru, Alexandru Bucur

**Affiliations:** 1 Faculty of Medicine, “Carol Davila” University of Medicine and Pharmacy, 050474 Bucharest, Romania; ioana.poiana@drd.umfcd.ro (I.R.P.); razvan-ionut.popescu@drd.umfcd.ro (R.-I.P.); silviu.pituru@umfcd.ro (S.-M.P.); alexandru.bucur@umfcd.ro (A.B.); 2 Department of Endocrinology, National Institute of Endocrinology C. I. Parhon, 011853 Bucharest, Romania; 3 Department of Urology, “Prof. Dr. Th. Burghele” Clinical Hospital, 050659 Bucharest, Romania

**Keywords:** cone-beam computed tomography, low bone mass, osteoporosis, osteopenia

## Abstract

Introduction: Cone-beam computed tomography (CBCT) is widely used in the preoperative qualitative and quantitative assessment of dental implant sites, offering dimensional accuracy, spatial resolution, gray density, and contrast comparable to those of classical CT scan, yet with disputable ability to determine bone mass density. Materials and methods: A systematic review of the literature was performed using the PubMed and SCOPUS databases, with terms referring to low bone mass and cone-beam computed tomography (CBCT). Results: Sixteen studies were included in the review. The results show different perspectives, but the evidence favors the use of CBCT, combined with dual-energy X-ray absorptiometry bone density scan (DXA) evaluation, for the assessment of the osteoporosis status of the aging population and, more specifically, in postmenopausal women. Radiographic density (RD) values of the dens and the left part of the first cervical vertebra show the strongest correlation coefficients and the highest sensitivity, specificity, and accuracy for predicting osteoporosis (OP) in the lumbar vertebrae and the femoral neck. Conclusions: Our review suggests the potential of CBCT as a screening tool for patients with low bone mass using different radiomorphometric indices. Linear measurements of the inferior mandibular cortex were lower in osteoporotic individuals, indicating the perspective of CBCT also as a diagnostic tool for this disease.

## 1. Introduction

Cone-beam computed tomography (CBCT) is frequently used in the preoperative qualitative and quantitative assessment of implant sites. It is preferred due to its relatively low cost and reduced radiation dose [1,2]. Low bone mass, diagnosed as osteopenia or osteoporosis (OP), is evaluated using dual-energy X-ray absorptiometry (DXA scan) and quantitative CT scan (QCT) [3].

There are a few studies showing the interactions between osteoporosis conditions and implant survival, and it is not known whether osteoporosis increases implant failure [4,5]. However, there is evidence indicating that implants installed in low-density bone tissues present a higher risk of failure [6].

CBCT offers dimensional accuracy, spatial resolution, gray density, and contrast comparable with those of a classical CT scan, but has debatable capability for the determination of bone mass density, as it does not offer bone quantity values, only gray density values [7]. Many studies have analyzed the relationship between CBCT-based gray density values (GDV) and Hounsfield Units (HU)-based CT values. The in vitro studies are controversial, because while Casetta and colleagues [8] demonstrated a linear correlation between VV and HU using a conversion rate in vitro and Chennoju [9] and colleagues provided an effective technique for reliably calculating the density using GDV, Varshowsaz and colleagues [10] showed that CBCT was not reliable for tissue density assessment.

It is debatable whether CBCT dental measurement can be used to assess bone mineral density, as it usually has a low resolution (i.e., 0.3–0.4 mm), causing serious blur in trabecular structures, which typically have a bone thickness of around 0.1 mm. The use for the analysis of bone mineral density is sustained by the CBCT information regarding trabecular patterns, such as the density and regularity of the bone structures, and structure-relevant features based on fractal analysis that can still provide discriminative information for separating different trabecular patterns despite the unavailability of detailed trabecular structures as a result of the low resolution used in clinical data. At the same time, gray values are inconsistent across different CBCT machines, and standardization may be necessary [11]. Another study demonstrated that artifacts in CBCT can interfere with the accurate conversion of density values into HUs, affecting the quantitative density measurements obtained with CBCT [12]. Determining the 3D mandibular osteoporosis index (MOI) for qualitative and quantitative assessment of the mandibular cortex has demonstrated sensitivity and specificity in distinguishing osteoporosis from normal bone mass density, with good predictive value.

Additionally, in vitro studies [13] have demonstrated the usefulness of the bone mass of the medial mandibular condyle (BDMC) for predicting high risk of osteoporosis, using specific cutoff values with a moderate correlation between BDMC and the mental index (MI). Additionally, trabecular bone structures can be accurately quantified by clinical dental CBCT in vitro, demonstrating a strong correlation with the results obtained by microCT [14]. At the same time, the accuracy of microCT for evaluating trabecular thickness and bone volume after subchondroplasty is questionable, as shown by a recent study published in 2023 [15].

The present study aims to offer a comprehensive review of the literature regarding the utility of CBCT for diagnosing low bone mass.

## 2. Materials and Methods

This systematic review was carried out according to the Preferred Reporting Items for Systematic Reviews and Meta-Analyses (PRISMA) criteria [16], detailed in the Appendix. The studies were included in the review with the aim of appreciating the diagnostic capability of CBCT imaging for the detection of low bone mass. A comprehensive electronic literature search was performed primarily using the PubMed and SCOPUS databased, as well as clinicaltrials.gov and the gray literature. Duplicate references were removed. The following keywords were used: (i) CBCT and osteoporosis; (ii) CBCT and osteopenia; and (iii) CBCT and low bone mass.

**Figure 1:**
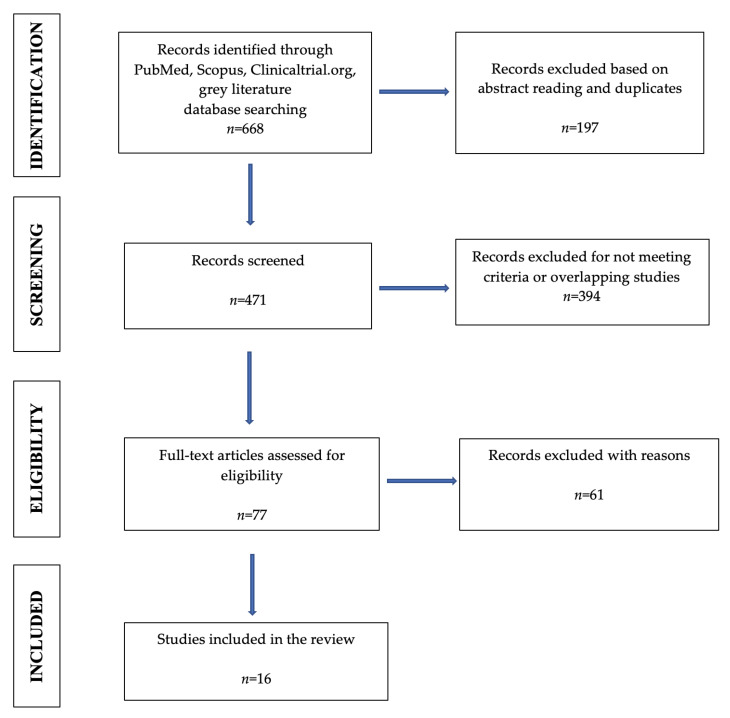
Flow diagram of the literature search and selection criteria adapted from the Preferred Reporting Items for Systematic Reviews and Meta-Analyses (PRISMA).

The PRISMA flow diagram is shown in Figure 1.

### 2.1. Inclusion Criteria

The reference images for bone mineral density were DXA images of the proximal femur or DXA images of the lumbar spine. The period for publication of the articles was January 2010–March 2022 in all databases. All studies included adult human patients with no differentiation on the basis of sex.

### 2.2. Exclusion Criteria

We excluded: (1) reviews, letters to the editor, case reports, book chapters, conference abstracts, and commentaries; (2) studies without reference imaging (DXA); and (3) experimental studies in vitro or in vivo using animal models.

For all included studies, the following features were registered: study characteristics (authors, year), group characteristics (case group, control group, mean age), intervention characteristics (index test, reference standard, methods), and outcome (main conclusions). The screening of the articles was conducted in duplicate. References cited within articles were also searched for relevance to the topic. The results were summarized, and the most significant results are discussed in the remainder of this article.

## 3. Results

Descriptions of the sixteen studies included in the review, with their main findings and limitations, are presented in Table 1.

The most used measurement instruments were the cortical indexes, specifically, CTI—cortical index (I, inferior, S, superior), with a total of 258 of patients (5 studies out of the 16 included). The main problem with the aforementioned studies is that a correlation was only found between normal BMD and osteoporosis (meaning lower BMD than osteopenia). None of the studies managed to demonstrate a correlation with lower BMD (Koh and colleagues demonstrated an important BMD difference between the two groups from −0.24 DS to −3.2 DS, while Mostafa and colleagues reported the difference as being even bigger, from +0.5 DS to −3.28 DS) [11,28]. Almost all of the studies included only normal BMD and osteoporosis patients.

Mathematical analysis of the fractal dimension (FD) was found in three of the included studies, one of which also included the largest number of patients [1]. The same study showed a significant difference between normal BMD and osteoporosis via mandibular FD analysis, but with low accuracy and reliability (AUC = 0.644, *p* = 0.008). The FD mandibular analysis of Mostafa and colleagues also showed a correlation between FD and lumbar spine BMD (r = 0359, *p* < 0.005) [11]. At the same time, Güngör and colleagues showed a significant positive correlation (r = 0.23, *p* < 0.05) between FD analysis and BMD when the FD measurement was performed at the right condyle, among the four separate areas of the jawbone analyzed (maxilla and left condyle) [23].

Shokri and colleagues analyzed only the gray values between the two groups, with gray values of maxillary tuberosity lower than 298 showing a 66% to 67% accuracy for the prediction of low BMD. This was the only study to analyze the gray value between normal BMD and lower BMD, with the radiographic density being calculated as gray (voxel) values, but with a clear cutoff definition for the values [18].

The radiographic density (RD) of the posterior mandibula obtained on the basis of analysis using the gray values of the CBCT showed a significant difference between the BMD of the normal and osteoporotic group (*p* < 0.005), with RD being strongly affected by the presence of osteoporosis; meanwhile, osteopenic patients were included in the control group. With respect to bone height, this parameter was only modified with respect to age: old-age non-osteoporotic females showed a lower level of alveolar bone level younger non-osteoporotic females [19]. Meanwhile, Barngkgei and colleagues showed that if the RD of the whole bony area of the mandible is equal to or lower than the gray values of 867–900 or 829–838, osteoporosis can be expected (diagnosed based on the lumbar or femoral neck BMD). The same authors showed that RD, determined on the basis of gray (voxel) values in the cervical vertebrae, is more strongly correlated with lumbar and femoral neck T-scores [27]. Regarding the measurements performed at the cervical vertebrae, another study [25] also showed that CBCT-derived RD values of the dens and the left part of the first cervical vertebra had a strong correlation with high sensitivity and sensibility in the prediction of osteoporosis (*p* < 0.001). 

Two studies analyzed jawbone trabecular structure using microstructural parameters of trabecular thickness (Tb.Th), trabecular spacing (Tb.Sp), and volume fraction (bone volume/total volume; BV/TV) [14,22], with inconsistent results, demonstrating (1) higher TbSp in osteoporotic patients (*p* = 0.004) and (2) high accuracy of Tb.Th and BS/TV of the dens.

## 4. Discussion

The success of dental implants largely depends on the amount of local bone and its condition [19]. Osteoporosis can affect alveolar bone, which may be associated with various types of dental work, especially dental implantation [29]. CBCT can play a great role in predicting patients with osteoporosis, a disease that might decrease the success rate of implantation [27]. Additionally, CBCT can represent an important adjuvant tool for the diagnosis of low bone mass, aiding in the screening process, especially in the elderly population.

In this review, we included 16 studies that evaluated specific indexes and measurements on the basis of CBCT in order to determine BMD via DXA scan to assess the possible role of CBCT dental evaluation in the screening and diagnosis of low bone mass.

Some studies have also been performed in which the Klemetti index and the mandibular cortical index (MCI) were evaluated as a means of qualitative assessment of BMD in dental imaging [30]. The evaluation of the MCI using panoramic reconstruction of CBCT showed a moderate sensitivity of 63.1% for a 5 mm thick slice and a specificity of 62.5% for a 25 mm one in a study published in 2018 that assessed 54 postmenopausal women [31]. At the same time, another study stated that the Klemetti index should not be used to assess osteoporosis on CBCT cross-sectional slices [31].

An earlier study published in 2011 [28] showed that the inferior and superior computed tomography mandibular indexes (CTIs) obtained via CBCT demonstrated significant differences between the normal and osteoporotic groups (*p* < 0.05) in 42 women. In a smaller study, CTIs were also moderately associated with osteoporosis, with a sensitivity of between 60 and 80%, and a specificity of 57.1–70% [24]. Considering the 3 mm mandibular cortical width on CBCT (CTMI) at the mental foramen as the cutoff threshold for densitometric evaluation, as reported in some studies, the mean value for osteoporotic patients was 2.33 mm compared to 3.22 for normal BMD patients [28], but this was not statistically significant [24].

Another study published in 2017 [21] showed that the mean values of CTIs were significantly lower in the osteoporotic group compared to in patients with normal bone mass, and even when compared to patients with osteopenia. At the same time, mean CTMI values were also lower in patients with osteoporosis compared to the other two groups (*p* = 0.000), and the mean values were lower than the 3 mm cutoff (2.93 mm for osteoporotic patients compared to 3.68 mm and 4.24 mm in osteopenia and normal BMD patients, respectively). In [21], the authors also observed statistically significant differences in the mean values of CTIs between normal BMD and osteopenia, but only for the superior index.

A systematic review aiming to evaluate the capability of CBCT in identifying patients with low bone mass was performed, including six studies published prior to 2016, which stated that only an endorsement could be made due to the low availability of studies [32]. Three of the included studies used the same sample of 38 postmenopausal women, among a total of 220 patients [32]. Three of the included studies showed that osteoporotic patients’ mandibular cortical measurements were lower [32]. Güngör et al. (2016) [23] also showed a CTMI lower than 3 mm in osteoporotic patients (2.76 mm), while values higher than 3 mm were obtained for the control group. Using radiographic density measured using gray values, four studies showed different values between low and normal bone mass, with excellent sensitivity and specificity being shown in only one study [32], potentially because of the different sites used, in this case, the C1 and C2 vertebrae and odontoid process [32]. In the same study, the density value for the mandibular body of the whole bony area on CBCT gave a general impression of the status of the femoral neck bone density on DXA. One of the most important observations was that the C1 and C2 analyses showed excellent accuracy in diagnostic tests when distinguishing patients with low BMD from those with normal BMD [32].

At the same time, using fractal dimension (FD) measurements to differentiate between low and normal bone mass showed a statistically significant correlation with the mandibular trabecular bone, but not with the vertebrae in a study published in 2022 [1], but with low accuracy (area under the curve of 0.64). At the same time, a meta-analysis regarding the use of fractal analysis in dental radiology for osteoporosis detection stated that FD could not be used to identify osteoporotic patients [33].

In 2020, the first test study was published that analyzed the accuracy of both qualitative and quantitative indices on CBCT for identifying low-BMD patients using a predictive model for the identification of osteoporotic patients on the basis of CBCT measurements and age [2]. The authors [2] compared over 50 osteoporotic patients with a similar number of patients with normal BMD, showing that mandibular cortical width (CTMI) was significantly lower in women with osteoporosis, and also, that postmenopausal women with osteoporosis were 8 times more likely to have CTMI thinner than 2.75 mm. A positive correlation was found with the BMD of the lumbar spine, femoral neck, and total hip, with a statistically significant association between the visual analysis of cortical quality and BMD [2]. The model had a good predictive ability, with an area under the ROC curve of 0.8 [2].

Other mandibular indices were evaluated in a single study, published in 2020 [17], in 48 postmenopausal women, including symphysis (S), a cross-sectional image equidistant from the centers of the right and left mental foramina (MF), 10 mm anterior to the MF, molar (M), 10 mm posterior to the MF, and posterior (P), 25 mm posterior to the MF, which are indices that are similar to the mandibular cortical width used in panoramic radiographs, but in different locations in the mandible. In [17], the authors showed that the M and P indices could be useful for identifying low BMD (*p* < 0.001), with a sensitivity of 75%, a moderate specificity of 62.5–68.7%, and an AUC of 0.74 and 0.69, respectively [17], with values below 3 mm as the cutoff stated in other studies.

Considering the same BMD, one study assessed the gray values on CBCT in different parts of the maxilla and mandible and compared them with DXA scans in the lumbar spine and femoral neck in 61 women, revealing a significant correlation between the T-score of the femoral neck and the gray values of different parts of the maxilla [18]. Furthermore, a significant correlation was noted between the T-score of the lumbar spine and the gray values of the anterior maxilla and maxillary tuberosity. The authors state that a gray value lower than 298 at the maxillary tuberosity can help distinguish patients with osteoporosis from normal individuals, with an accuracy of 66% to 67% [18].

In a study comparing women with normal bone density with those with osteoporosis [24], mandibular bone width, and cortical quality were significantly lower in the latter group. In another study, published in 2018, bone density evaluated by CBCT was strongly affected by the presence of osteoporosis, and it decreased, as the mean for osteoporotic females aged 50 years and above was lower than the mean for non-osteoporotic females aged 50 years and above [19]. In the same study, the mandibular posterior area density using the gray value of CBCT was a very good indicator for osteoporotic patients, whereas alveolar bone height measurement was unreliable for BMD conditions, being more affected by the aging factor.

Some studies have demonstrated that the cortical measurements detected on the panoramic images on CBCT might be useful for identifying younger postmenopausal women with a low BMD or osteoporosis [34]. Right and left mandibular radiomorphometric indexes and CT values in osteoporosis patients were significantly lower than measurements in osteopenia and controls in a study with 90 patients. Positive correlations were observed between spine bone mineral density measurements on DXA and right and left mandibular CT values [23].

At the same time, it was demonstrated that bone density at the cervical vertebrae (C1 and C2) evaluated with CBCT could be used to correctly assess osteoporosis [2,3,24], and a correlation of bone mass at the vertebrae with osteoporosis was also performed regarding the gray areas [19].

Only one study [26] evaluated the mandibular ridge using various measurements in 45 edentulous women and assessed the relationship with BMD on DXA scan, with no correlation being found between the general BMD and the size of the mandibular ridge; these results corroborated those of previous older studies.

A study published in 2023 [14] that included 18 OP patients and 27 control patients, as well as patients with systemic diseases like diabetes/advanced chronic kidney disease, showed lower BV/TV (bone volume fraction) and Tb.Th (trabecular thickness) in the OP group, and higher Tb.S (trabecular separation) values (*p* = 0.004). These results show that trabecular bone quality is negatively affected by OP, and cortical and trabecular bone parts must be investigated separately and combined with patients’ clinical symptoms. The authors stated that CBCT could be suitable for the microstructural evaluation of trabecular bone, and the mandible can carry valuable data for this purpose [14].

The use of CBCT in combination with DXA evaluation could surely be helpful in assessing osteoporosis status in the aging population and, more specifically, in peri- or postmenopausal women.

Sixteen studies were included in this paper in which different radiomorphometric indicators were proposed for low BMD on CBCT scans. In our review, RD values of the dens and the left part of the first cervical vertebra showed the strongest correlation coefficients and the highest sensitivity, specificity, and accuracy for predicting OP in the lumbar vertebrae and the femoral neck. The cervical vertebrae RD values strongly correlate with lumbar and femoral neck T-scores on DXA evaluation. Additionally, linear measurements of the inferior mandibular cortex were lower in osteoporotic individuals, indicating the potential of CBCT as a diagnostic tool for this disease. 

## 5. Conclusions

Defective bone formation and increased bone resorption can lead to deterioration in the microstructure of trabecular bone, resulting in increased bone fragility and risk of fracture, making the presence of low bone mass a challenge for implantology.

Although many techniques are available for assessing the mineral density of bone, the most widely used is based on X-ray absorptiometry in bone. In many countries, osteoporosis is underdiagnosed. At the same time, the majority of fragility fractures are diagnosed in patients with osteopenia.

The present systematic review evaluated the available data concerning the diagnostic accuracy of CBCT for osteoporosis screening by differentiating individuals with low BMD from those individuals with normal BMD. CBCT scans are increasingly being used in stomatology, especially for implant planning in edentulous patients, a potential population for osteoporosis. There are a few studies showing the interactions between osteoporosis conditions and implant survival, which is why CBCT can be a powerful tool for predicting implant success. At the same time, osteoporotic patients are candidates for pharmacological treatment that could further increase the chances of implant success.

In cases where dental specialists prescribe CBCT, this could play a promising role in identifying patients with low BMD and, perhaps, the relationship of low BMD with implant survival.

## Figures and Tables

**Table 1:** Main findings and limitations of the studies included in this review.

Nr. Crt	Study Details	Year	No. Pax.	Sex	Type of Patients	Measurement (Quantified)	Relevant Findings	Limitations
1.	(Bilgili and Üçok, 2023) [14]	2023	18/27	W	OP/Normal	Microstructural parameters of trabecular thickness (Tb.Th), trabecular spacing (Tb.Sp), and volume fraction (bone volume/total volume, BV/TV)	Trabecular spacing was higher in OP compared to the control (*p* = 0.004). Tb.Th and BV/TV values were higher in the control group.	Low number of patients; BV/TV and Tb.Th correlations in OP versus the control group are not statistically significant.
2.	(Carvalho et al., 2022) [1]	2022	103	W	Normal/OP	FD values of the second vertebra and mandible	Different FD values between women with normal BMD and those with osteoporosis at mandibular sites, but with low accuracy and reliability (AUC of 0.644).	FD measurements were performed on a 2-dimensional reconstruction.
3.	(Barra et al., 2021) [17]	2020	48	W	Normal/ osteopenia/OP	Symphysis (S) cross-sectional image equidistant from the centers of the right and left mental foramina (MF); anterior (A): 10 mm anterior to the MF; molar (M): 10 mm posterior to the MF; and posterior (P): 25 mm posterior to the MF	All indexes were lower in OP/osteopenia compared to patients with normal BMD (*p* < 0.001).	Low specificity (37.5%), high sensitivity.
4.	(de Castro et al., 2020) [2]	2020	52/51	W	Normal BMD/OP	Mandibular cortical width (MCW)	Postmenopausal women with osteoporosis were 8 times more likely to have the cortex classified as C3, and 2.4 times more likely to have MCW thinner than 2.75 mm.	Reliability of the qualitative index, low reproducibility. Moderate intraobserver agreement (kappa = 0.6), moderate interobserver agreement for the qualitative analysis (kappa = 0.4).
5.	(Shokri et al., 2019) [18]	2019	61	W	N/osteopenia/OP	CBCT GV of the maxilla	The clear definition of the cut-off points of GV for different parts of the jaw; significant correlations were found between the T-scores of the femoral neck and the GV of cancellous bone (*p* = 0.042) and those of cancellous and cortical bones (*p* = 0.045) segments at the site of the maxillary incisors, the cancellous and cortical bone segments at the site of the maxillary premolars (*p* = 0.043), and the cancellous bone segment (*p* = 0.003) and the cancellous and cortical bone segments (*p* = 0.001) at the maxillary tuberosity. Significant correlations were found between the T-scores of the lumbar spine and the GV of the cancellous bone segment of maxillary incisors (*p* = 0.046) and the cancellous bone segment (*p* = 0.008) and the cancellous and cortical bone segments (*p* = 0.003) at the maxillary tuberosity. Excellent predictive value of the variables.	Calculating the RD as gray (voxel) values; densities of different parts of the mandible were not correlated with DXA bone density; the osteoporotic group also included an osteopenia group.
6.	(Albayati, Saliem and Al Nakib, 2018) [19]	2018	60	W	Normal BMD/OP	Posterior mandibular first molar radiographic density (RD) and alveolar bone height	RD in the posterior mandible first molar area is significantly affected in OP patients; this can be used as a predictor for the presence of OP using CBCT.	Patients with osteopenia were included in the control group; patients were not divided by their dental condition
7.	(Kato et al., 2019) [20]	2018	54	W	Normal/low BMD	Mandibular bone cortex	The panoramic reconstruction of CBCT with 25 mm slice thickness seems to be the most accurate with a Sn of 52.6% and Sp of 62.5% and AUC of 57.6%.	Low sensitivity and specificity.
8.	(Brasileiro et al., 2017) [21]	2017	60	W	Normal/osteopenia/OP	CTI, CTMI	Mean values of CTMI, CTI (S), and CTI (I) were lower in the osteoporosis group than in osteopenia and normal patients (*p* < 0.05).	No statistically significant difference in CTI (I) between normal BMD and osteopenia group.
9.	(Barngkgei et al., 2016) [22]	2016	38	W	Normal BMD/OP	Trabecular bone structure: trabecular thickness (Tb.Th), trabecular separation (Tb.S), bone volume fraction (BS/TV), specific bone surface (BS/TV), and connective density	The first study to evaluate TBS, Tb.Th and BS/TV of the dens, with high accuracy in predicting osteoporosis (*p* = 0.014); Relatively large voxel size.	No statistically significant in almost all jaw-bone variables.
10.	(Güngör, Yildirim and Çevik, 2016) [23]	2016	90	W, M	Normal/osteopenia/OP	CTI (S), CTI (I), CTMI, CT values, FD, HA	OP caused significant changes in radio morphometric indexes and CT values in the jaw bones; left maxilla FD measurements in osteoporosis patients were significantly lower than in the control (*p* ≤ 0.05) and osteopenia (*p* ≤ 0.05) groups; HA measurements from left maxilla, left & right mandible significantly lower in the OP group.	No significant differences In the index measurements in the osteopenia and control group; a correlation only of CTMI index with the BMD of the femoral head.
11.	(Mostafa, Arnout and Abo El-Fotouh, 2016) [11]	2016	50	W	Normal BMD/OP	CTI, CTMI, CTCI, FD	Positive correlation between CTMI and CTI with lumbar spine BMD ensured by DXA (*p* < 0.001); significant negative correlation between CTCI scores and BMD of the lumbar spine.	Only normal BMD and OP patients were included (no evaluation of osteopenia).
12.	(Geibel, Löffler and Kildal, 2016) [24]	2016	16	W, M	Normal BMD/OP	CTI (computed tomography mandibular index, S-superior, I-inferior), CTMI (computed tomography mental index)	CTMI values located < 3.0 mm (80% sensitivity and 57.1% specificity) can be suggestive of OP for the female subject group.	Low number of patients; low specificity; only 6 patients with osteoporosis; CBCT was up to 2 years older than DXA measurements.
13.	(Barngkgei, Joury and Jawad, 2015) [25]	2015	38	W	Normal BMD/OP	RD values of the first and second vertebrae	RD values of the dens and the left part of the first cervical vertebra showed the strongest correlation coefficients (r = 0.7, 0.6; *p* < 0.001) and the highest sensitivity (76.9%, 70%), specificity (92%, 92.9%), and accuracy (90.8%, 86.4%) in predicting OP in the lumbar vertebrae and the femoral neck; the cervical vertebrae RD values are more strongly correlated with lumbar and femoral neck T-scores.	RD calculated as gray (voxel) values.
14.	(Springe et al., 2014) [26]	2014	45	W	N/osteopenia/OP	Mandibular residual ridge (height, width)	The statistically significant relationship between BMD and two width measurements performed in the midline edentulous women.	No statistically significant relationship
15.	(Barngkgei, Al Haffar and Khattab, 2014) [27]	2014	38	W	Normal BMD/OP	RD of the mandible	Lumbar vertebrae and femoral neck OP can be predicted with high accuracy from the RD value of the body of the mandible by using CBCT.	Calculating the RD as gray (voxel) values, the cervical vertebrae RD values are more strongly correlated with lumbar and femoral neck T-scores.
16.	(Koh and Kim, 2011) [28]	2011	42	W	Normal BMD/OP	CTI (S), CTI (I), CTMI, CTCI	CTI (S) and CTI (I) were significantly different between the normal and osteoporotic groups (*p* < 0.05).	No correlation between the intra-observer agreement regarding CTMI.

W—women; M—men; OP—osteoporosis; BMD—bone mineral density; N—normal; MCW—mandibular cortical width; AUC—area under the curve; BS/TV—bone volume fraction; CTI—cortical index (I, inferior; S, superior); RD—radiographic density; Tb.S—trabecular separation; Tb.Th—trabecular thickness; FD—fractal dimension; HA—histogram analysis; CTCI—computed tomography cortical index.

## Data Availability

All information included in this review is documented with relevant references.
